# 2-(4-Chloro­phen­yl)-2-oxoethyl benzoate

**DOI:** 10.1107/S160053681102383X

**Published:** 2011-06-25

**Authors:** Hoong-Kun Fun, Tara Shahani, B. Garudachari, Arun M. Isloor, M. N. Satyganarayan

**Affiliations:** a X-ray Crystallography Unit, School of Physics, Universiti Sains Malaysia, 11800 USM, Penang, Malaysia; b Organic Electronics Division, Department of Chemistry, National Institute of Technology-Karnataka, Surathkal, Mangalore 575 025, India; c Department of Physics, National Institute of Technology-Karnataka, Surathkal, Mangalore 575 025, India

## Abstract

In the title compound, C_15_H_11_ClO_3_, the dihedral angle between the aromatic rings is 84.29 (8)°. In the crystal, mol­ecules are linked by weak C—H⋯π inter­actions.

## Related literature

For applications of phenacyl benzoate derivatives, see: Rather & Reid (1919 ▶); Litera *et al.* (2006 ▶); Huang *et al.* (1996 ▶); Gandhi *et al.* (1995 ▶). For related structures, see: Ogata *et al.* (1987 ▶); Wan *et al.* (2006 ▶); Zhang *et al.* (2006 ▶). For reported melting-point details, see: Le *et al.* (2009 ▶). For bond-length data, see: Allen *et al.* (1987 ▶).
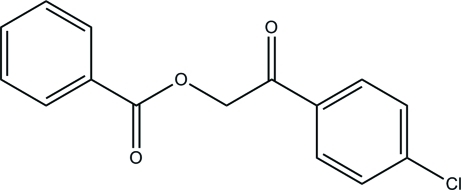


## Experimental

### 

#### Crystal data

C_15_H_11_ClO_3_
*M* *_r_* = 274.69
Monoclinic, 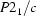

*a* = 8.1955 (9) Å
*b* = 10.8717 (12) Å
*c* = 16.5420 (15) Å
β = 117.816 (4)°
*V* = 1303.6 (2) Å^3^
*Z* = 4
Mo *K*α radiation
μ = 0.29 mm^−1^
*T* = 296 K
0.34 × 0.19 × 0.19 mm

#### Data collection

Bruker SMART APEXII CCD diffractometer
Absorption correction: multi-scan (*SADABS*; Bruker, 2009 ▶) *T*_min_ = 0.908, *T*_max_ = 0.948
11201 measured reflections
4052 independent reflections
2720 reflections with *I* > 2σ(*I*)
*R*_int_ = 0.021

#### Refinement

*R*[*F* ^2^ > 2σ(*F* ^2^)] = 0.044
*wR*(*F* ^2^) = 0.126
*S* = 1.03
4052 reflections
172 parameters
H-atom parameters constrained
Δρ_max_ = 0.31 e Å^−3^
Δρ_min_ = −0.49 e Å^−3^

### 

Data collection: *APEX2* (Bruker, 2009 ▶); cell refinement: *SAINT* (Bruker, 2009 ▶); data reduction: *SAINT*; program(s) used to solve structure: *SHELXTL* (Sheldrick, 2008 ▶); program(s) used to refine structure: *SHELXTL*; molecular graphics: *SHELXTL*; software used to prepare material for publication: *SHELXTL* and *PLATON* (Spek, 2009 ▶).

## Supplementary Material

Crystal structure: contains datablock(s) global, I. DOI: 10.1107/S160053681102383X/hb5918sup1.cif

Structure factors: contains datablock(s) I. DOI: 10.1107/S160053681102383X/hb5918Isup2.hkl

Supplementary material file. DOI: 10.1107/S160053681102383X/hb5918Isup3.cml

Additional supplementary materials:  crystallographic information; 3D view; checkCIF report

## Figures and Tables

**Table 1:** Hydrogen-bond geometry (Å, °) *Cg*2 is the centroid of the C10–C15 ring.

*D*—H⋯*A*	*D*—H	H⋯*A*	*D*⋯*A*	*D*—H⋯*A*
C8—H8*A*⋯*Cg*2^i^	0.97	2.96	3.4952 (17)	116

Symmetry code: (i) 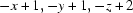.

**Fig. 1.**
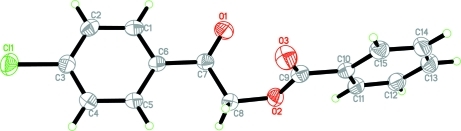
The molecular structure of the title compound, showing 30% probability displacement ellipsoids.

**Fig. 2.**
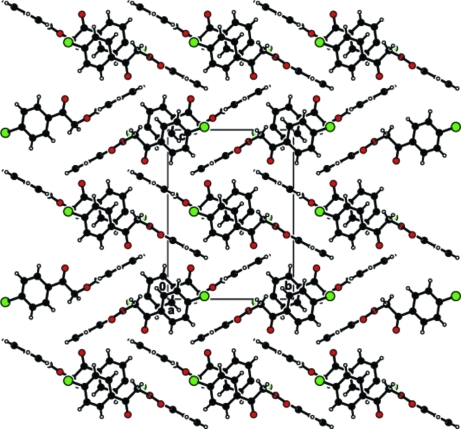
The crystal packing of the title compound (I).

## supplementary crystallographic information

### Comment

Phenacyl benzoate derivatives are very important in identification of organic acids (Rather & Reid, 1919) as they undergo photolysis in neutral and mild conditions (Litera *et al.*, 2006). They find applications in the field of synthetic chemistry for the synthesis of oxazoles, imidazoles (Huang *et al.*, 1996) and benzoxazepine (Gandhi *et al.*, 1995). We hereby report the crystal structure of the title compound, (I).

The asymmetric unit of title compound is shown in Fig. 1. The dihedral angle between the phenyl (C10–C15) ring and the chloro-substituted phenyl (C1–C6) ring is 84.29 (8)°. The bond lengths (Allen *et al.*, 1987) and angles are within normal ranges and are comparable to those closely related structures (Ogata *et al.*, 1987; Wan *et al.*, 2006; Zhang *et al.*, 2006).

In the crystal (Fig. 2), there are no classical hydrogen bonds but stabilization is provided by weak C—H···π (Table 1) interactions, involving the *Cg*2 (C10–C15) ring.

### Experimental

A mixture of benzoic acid (1.0 g, 0.0081 mol), potassium carbonate (1.23 g, 0.0089 mol) and 2-bromo-1-(4-chlorophenyl) ethanone (1.81 g, 0.0081 mol) in dimethylformamide (10 ml) was stirred at room temperature for 2 h. On cooling, colorless needle-shaped crystals of 2-(4-chlorophenyl)-2-oxoethyl benzoate begin to separate out. These were collected by filtration and recrystallized from ethanol to yield colourless blocks of (I). Yield: 2.10 g, 93.7%, *Mp*: 119–120 °C (Le *et al.*, 2009).

### Refinement

All the H atoms were positioned geometrically [C–H = 0.93–0.97 Å] and were refined using a riding model, with *U*_iso_(H) =1.2. *U*_eq_(C).

### Crystal data

**Table:**

C_15_H_11_ClO_3_	*F*(000) = 568
*M**_r_* = 274.69	*D*_x_ = 1.400 Mg m^−^^3^
Monoclinic, *P*2_1_/*c*	Mo *K*α radiation, λ = 0.71073 Å
Hall symbol: -P 2ybc	Cell parameters from 2797 reflections
*a* = 8.1955 (9) Å	θ = 2.3–27.1°
*b* = 10.8717 (12) Å	µ = 0.29 mm^−^^1^
*c* = 16.5420 (15) Å	*T* = 296 K
β = 117.816 (4)°	Block, colourless
*V* = 1303.6 (2) Å^3^	0.34 × 0.19 × 0.19 mm
*Z* = 4	

### Data collection

**Table:**

Bruker SMART APEXII CCD diffractometer	4052 independent reflections
Radiation source: fine-focus sealed tube	2720 reflections with *I* > 2σ(*I*)
graphite	*R*_int_ = 0.021
φ and ω scans	θ_max_ = 30.8°, θ_min_ = 2.3°
Absorption correction: multi-scan (*SADABS*; Bruker, 2009)	*h* = −11→11
*T*_min_ = 0.908, *T*_max_ = 0.948	*k* = −15→15
11201 measured reflections	*l* = −20→23

### Refinement

**Table:**

Refinement on *F*^2^	Primary atom site location: structure-invariant direct methods
Least-squares matrix: full	Secondary atom site location: difference Fourier map
*R*[*F*^2^ > 2σ(*F*^2^)] = 0.044	Hydrogen site location: inferred from neighbouring sites
*wR*(*F*^2^) = 0.126	H-atom parameters constrained
*S* = 1.03	*w* = 1/[σ^2^(*F*_o_^2^) + (0.0504*P*)^2^ + 0.2649*P*] where *P* = (*F*_o_^2^ + 2*F*_c_^2^)/3
4052 reflections	(Δ/σ)_max_ < 0.001
172 parameters	Δρ_max_ = 0.31 e Å^−^^3^
0 restraints	Δρ_min_ = −0.49 e Å^−^^3^

### Special details

**Table:**

Geometry. All e.s.d.'s (except the e.s.d. in the dihedral angle between two l.s. planes) are estimated using the full covariance matrix. The cell e.s.d.'s are taken into account individually in the estimation of e.s.d.'s in distances, angles and torsion angles; correlations between e.s.d.'s in cell parameters are only used when they are defined by crystal symmetry. An approximate (isotropic) treatment of cell e.s.d.'s is used for estimating e.s.d.'s involving l.s. planes.
Refinement. Refinement of *F*^2^ against ALL reflections. The weighted *R*-factor *wR* and goodness of fit *S* are based on *F*^2^, conventional *R*-factors *R* are based on *F*, with *F* set to zero for negative *F*^2^. The threshold expression of *F*^2^ > σ(*F*^2^) is used only for calculating *R*-factors(gt) *etc*. and is not relevant to the choice of reflections for refinement. *R*-factors based on *F*^2^ are statistically about twice as large as those based on *F*, and *R*- factors based on ALL data will be even larger.

### Fractional atomic coordinates and isotropic or equivalent isotropic displacement parameters (Å^2^)

**Table:**

	*x*	*y*	*z*	*U*_iso_*/*U*_eq_	
Cl1	0.98407 (8)	1.29129 (4)	1.01748 (4)	0.07410 (18)	
O1	0.41826 (16)	0.81706 (12)	0.81726 (7)	0.0648 (3)	
O2	0.41542 (15)	0.64299 (10)	0.93059 (7)	0.0544 (3)	
O3	0.59892 (16)	0.55553 (13)	0.87990 (9)	0.0722 (4)	
C1	0.6218 (2)	1.03708 (15)	0.86536 (10)	0.0509 (3)	
H1A	0.5394	1.0250	0.8040	0.061*	
C2	0.7272 (2)	1.14249 (14)	0.89170 (12)	0.0545 (4)	
H2A	0.7170	1.2011	0.8486	0.065*	
C3	0.8484 (2)	1.15975 (14)	0.98327 (11)	0.0507 (3)	
C4	0.8651 (2)	1.07434 (15)	1.04787 (11)	0.0555 (4)	
H4A	0.9469	1.0874	1.1092	0.067*	
C5	0.7593 (2)	0.96902 (14)	1.02070 (10)	0.0505 (3)	
H5A	0.7701	0.9109	1.0642	0.061*	
C6	0.63658 (18)	0.94852 (13)	0.92902 (9)	0.0435 (3)	
C7	0.52322 (18)	0.83503 (14)	0.89703 (10)	0.0458 (3)	
C8	0.5435 (2)	0.74141 (15)	0.96859 (10)	0.0524 (4)	
H8A	0.6680	0.7087	0.9967	0.063*	
H8B	0.5243	0.7814	1.0159	0.063*	
C9	0.4586 (2)	0.55549 (15)	0.88590 (10)	0.0510 (3)	
C10	0.3128 (2)	0.46016 (14)	0.84710 (9)	0.0473 (3)	
C11	0.1476 (2)	0.47129 (15)	0.85064 (10)	0.0520 (3)	
H11A	0.1261	0.5397	0.8781	0.062*	
C12	0.0150 (2)	0.38051 (17)	0.81327 (11)	0.0622 (4)	
H12A	−0.0966	0.3888	0.8147	0.075*	
C13	0.0468 (3)	0.27865 (18)	0.77419 (12)	0.0688 (5)	
H13A	−0.0424	0.2174	0.7498	0.083*	
C14	0.2104 (3)	0.26665 (17)	0.77089 (12)	0.0706 (5)	
H14A	0.2317	0.1970	0.7445	0.085*	
C15	0.3436 (3)	0.35746 (15)	0.80651 (11)	0.0587 (4)	
H15A	0.4534	0.3496	0.8032	0.070*	

### Atomic displacement parameters (Å^2^)

**Table:**

	*U*^11^	*U*^22^	*U*^33^	*U*^12^	*U*^13^	*U*^23^
Cl1	0.0961 (4)	0.0549 (3)	0.0902 (4)	−0.0203 (2)	0.0593 (3)	−0.0167 (2)
O1	0.0575 (7)	0.0764 (8)	0.0440 (6)	−0.0114 (6)	0.0100 (5)	0.0018 (5)
O2	0.0573 (6)	0.0533 (6)	0.0590 (6)	−0.0116 (5)	0.0325 (5)	−0.0065 (5)
O3	0.0551 (7)	0.0808 (9)	0.0885 (9)	−0.0061 (6)	0.0399 (7)	−0.0124 (7)
C1	0.0525 (8)	0.0567 (9)	0.0423 (7)	0.0060 (7)	0.0212 (6)	0.0066 (6)
C2	0.0646 (9)	0.0483 (8)	0.0593 (9)	0.0064 (7)	0.0361 (8)	0.0109 (7)
C3	0.0559 (8)	0.0453 (7)	0.0615 (9)	−0.0017 (6)	0.0362 (7)	−0.0066 (7)
C4	0.0624 (9)	0.0564 (9)	0.0471 (8)	−0.0057 (7)	0.0251 (7)	−0.0062 (7)
C5	0.0583 (8)	0.0518 (8)	0.0404 (7)	−0.0027 (7)	0.0222 (6)	0.0035 (6)
C6	0.0434 (7)	0.0475 (7)	0.0411 (7)	0.0036 (6)	0.0210 (6)	0.0020 (6)
C7	0.0404 (7)	0.0535 (8)	0.0419 (7)	0.0010 (6)	0.0179 (6)	0.0008 (6)
C8	0.0554 (8)	0.0541 (8)	0.0462 (8)	−0.0102 (7)	0.0224 (7)	−0.0010 (7)
C9	0.0506 (8)	0.0537 (8)	0.0485 (8)	0.0010 (7)	0.0229 (7)	0.0053 (7)
C10	0.0526 (8)	0.0463 (7)	0.0405 (7)	0.0016 (6)	0.0196 (6)	0.0066 (6)
C11	0.0545 (8)	0.0520 (8)	0.0486 (8)	−0.0029 (7)	0.0233 (7)	0.0030 (7)
C12	0.0579 (9)	0.0679 (10)	0.0539 (9)	−0.0110 (8)	0.0203 (8)	0.0042 (8)
C13	0.0739 (12)	0.0609 (10)	0.0541 (10)	−0.0154 (9)	0.0151 (9)	0.0009 (8)
C14	0.0960 (14)	0.0496 (9)	0.0526 (10)	0.0016 (9)	0.0233 (10)	−0.0037 (8)
C15	0.0692 (10)	0.0547 (9)	0.0520 (9)	0.0092 (8)	0.0282 (8)	0.0051 (7)

### Geometric parameters (Å, °)

**Table:**

Cl1—C3	1.7362 (16)	C7—C8	1.510 (2)
O1—C7	1.2083 (17)	C8—H8A	0.9700
O2—C9	1.3492 (19)	C8—H8B	0.9700
O2—C8	1.4235 (18)	C9—C10	1.482 (2)
O3—C9	1.1996 (18)	C10—C15	1.385 (2)
C1—C2	1.378 (2)	C10—C11	1.388 (2)
C1—C6	1.390 (2)	C11—C12	1.382 (2)
C1—H1A	0.9300	C11—H11A	0.9300
C2—C3	1.383 (2)	C12—C13	1.367 (3)
C2—H2A	0.9300	C12—H12A	0.9300
C3—C4	1.373 (2)	C13—C14	1.373 (3)
C4—C5	1.379 (2)	C13—H13A	0.9300
C4—H4A	0.9300	C14—C15	1.384 (3)
C5—C6	1.3920 (19)	C14—H14A	0.9300
C5—H5A	0.9300	C15—H15A	0.9300
C6—C7	1.486 (2)		
			
C9—O2—C8	116.36 (12)	O2—C8—H8B	109.3
C2—C1—C6	121.10 (14)	C7—C8—H8B	109.3
C2—C1—H1A	119.5	H8A—C8—H8B	107.9
C6—C1—H1A	119.5	O3—C9—O2	123.48 (15)
C1—C2—C3	118.90 (14)	O3—C9—C10	125.06 (15)
C1—C2—H2A	120.6	O2—C9—C10	111.46 (13)
C3—C2—H2A	120.6	C15—C10—C11	119.52 (15)
C4—C3—C2	121.36 (15)	C15—C10—C9	118.75 (14)
C4—C3—Cl1	119.15 (13)	C11—C10—C9	121.72 (14)
C2—C3—Cl1	119.49 (13)	C12—C11—C10	119.86 (16)
C3—C4—C5	119.25 (15)	C12—C11—H11A	120.1
C3—C4—H4A	120.4	C10—C11—H11A	120.1
C5—C4—H4A	120.4	C13—C12—C11	120.41 (18)
C4—C5—C6	120.85 (14)	C13—C12—H12A	119.8
C4—C5—H5A	119.6	C11—C12—H12A	119.8
C6—C5—H5A	119.6	C12—C13—C14	120.06 (17)
C1—C6—C5	118.54 (14)	C12—C13—H13A	120.0
C1—C6—C7	119.12 (13)	C14—C13—H13A	120.0
C5—C6—C7	122.34 (13)	C13—C14—C15	120.43 (17)
O1—C7—C6	122.11 (14)	C13—C14—H14A	119.8
O1—C7—C8	120.59 (14)	C15—C14—H14A	119.8
C6—C7—C8	117.30 (12)	C14—C15—C10	119.69 (17)
O2—C8—C7	111.81 (12)	C14—C15—H15A	120.2
O2—C8—H8A	109.3	C10—C15—H15A	120.2
C7—C8—H8A	109.3		
			
C6—C1—C2—C3	−0.4 (2)	C6—C7—C8—O2	174.36 (12)
C1—C2—C3—C4	−0.1 (2)	C8—O2—C9—O3	2.5 (2)
C1—C2—C3—Cl1	179.31 (11)	C8—O2—C9—C10	−178.30 (12)
C2—C3—C4—C5	0.3 (2)	O3—C9—C10—C15	5.2 (2)
Cl1—C3—C4—C5	−179.14 (12)	O2—C9—C10—C15	−173.94 (13)
C3—C4—C5—C6	0.0 (2)	O3—C9—C10—C11	−174.87 (16)
C2—C1—C6—C5	0.7 (2)	O2—C9—C10—C11	5.9 (2)
C2—C1—C6—C7	−178.58 (13)	C15—C10—C11—C12	−0.4 (2)
C4—C5—C6—C1	−0.5 (2)	C9—C10—C11—C12	179.72 (14)
C4—C5—C6—C7	178.73 (14)	C10—C11—C12—C13	1.1 (2)
C1—C6—C7—O1	0.0 (2)	C11—C12—C13—C14	−0.8 (3)
C5—C6—C7—O1	−179.21 (15)	C12—C13—C14—C15	−0.3 (3)
C1—C6—C7—C8	−179.92 (13)	C13—C14—C15—C10	1.1 (3)
C5—C6—C7—C8	0.9 (2)	C11—C10—C15—C14	−0.7 (2)
C9—O2—C8—C7	79.04 (17)	C9—C10—C15—C14	179.19 (15)
O1—C7—C8—O2	−5.6 (2)		

### Hydrogen-bond geometry (Å, °)

**Table:**

Cg2 is the centroid of the C10–C15 ring.

**Table:**

*D*—H···*A*	*D*—H	H···*A*	*D*···*A*	*D*—H···*A*
C8—H8A···Cg2^i^	0.97	2.96	3.4952 (17)	116

Symmetry codes: (i) −*x*+1, −*y*+1, −*z*+2.
